# Genome Sequence of *Arthrobacter* Phage KNG13

**DOI:** 10.17912/micropub.biology.002088

**Published:** 2026-05-15

**Authors:** Kelci Grahmann, Chance Cecchine, Madison Rosales, Andrea Lozano, Katey Stoddard, Francene Wang, Nicholas Long, Benjamin Neuman, Sofia Sanchez, Miguel Bernal, Halle Brown, Mia Enciso, James Hunter, Ryan Le, Faye Selesi, Danielle Toscano, Joseph Urrabazo, Kristina Vaquera, Martin Villarreal, Nicholas Hernandez

**Affiliations:** 1 Biology, Health, and the Environment, The University of Texas at San Antonio, San Antonio, TX, US

## Abstract

Bacteriophage KNG13, a siphovirus, was isolated in San Antonio, Texas, USA, using *Arthrobacter globiformis* B-2979. KNG13 has a genome length of 15,768-bp and is assigned to actinobacteriophage cluster FE based on gene content.

**Figure 1. Plaque morphology of KNG13:** Clear, circular plaques of KNG13 on a 100 mm PYCa agar plate using an *Arthrobacter globiformis* lawn.

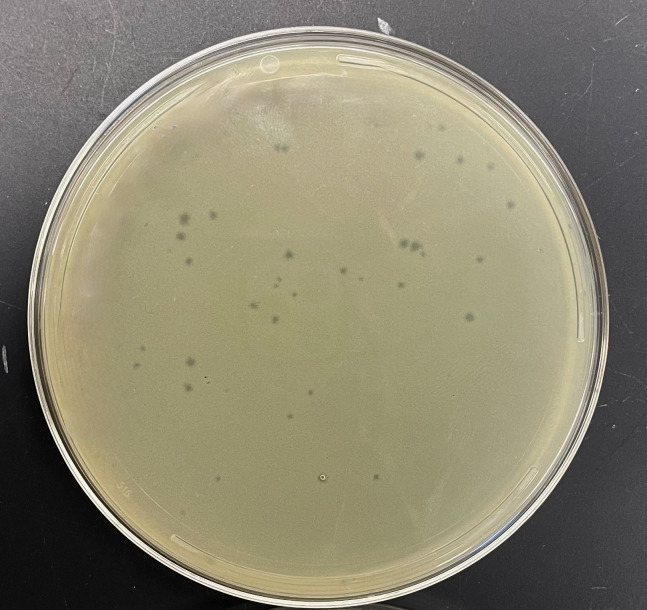



## Description

Bacteriophages are incredibly abundant and diverse, with multitude of applications ranging from phage therapy to biotechnology (Dedrick et al., 2019; Naureen et al., 2020; Rogovski et al., 2021). Here, we describe the isolation and characterization of a novel bacteriophage, KNG13. 

Phage KNG13 was extracted from mildly damp soil collected on a trail in San Antonio, Texas, USA (29.663765 N, 98.421426 W) when the ambient temperature was approximately 27°C. The soil sample was processed using standard protocols (Zorawik et al., 2024). Briefly, approximately 7 cm ^3^ of soil sample was suspended in 10 ml PYCa (peptone, yeast extract, calcium chloride, and dextrose) liquid medium and agitated in a shaking incubator for 2 hours. The suspension was then spun (2,000 x g for 10 minutes) and the supernatant filtered using a syringe filter (0.2-micron pore size). The filtrate was subsequently plated in PYCa top agar with *Arthrobacter globiformis* B-2979 and plates incubated at 30 ˚C for 48 hours. Phage KNG13, which forms plaques that are clear and approximately 1.5 mm in diameter (Figure 1), was purified by two rounds of selecting plaques and plating.

A lysate for KNG13 was prepared (2.8 x 10 ^9^ PFU/mL) and used to extract DNA with the Promega Wizard DNA kit. Phage DNA was then prepared for sequencing with the NEB Ultra FS kit and sequenced on an Illumina NextSeq 1000 (XLEAP-P1 kit), yielding 1,345,255 single-end 100 base reads. Raw sequencing reads were trimmed using cutadapt v4.7 (using the option: –nextseq-trim 30) and subsequently filtered with skewer v0.2.2 (using the options: -q 20 -Q 30 -n -l 50) prior to assembly. The genome was assembled using Newbler v.29 (Miller et al., 2010) and checked for completeness using Consed v2.9 (Gordon et al., 1998), resulting in an assembled genome of 15,768 base pairs with 7,815-fold coverage. The genome consisted of 67.6 % GC content, with 3’ single-stranded overhangs of a 5’CCACGTATACCGTCC.

The genome was annotated with DNA Master v5.23.6 (cobamide2.bio.pitt.edu) and PECAAN v20250130 (discover.kbrinsgd.org) (Rinehart et al., 2016), using Glimmer v3.02 (Delcher et al. 2007 ) and GeneMark v2.5p (Besemer and Borodovsky 2005) to predict genes and Starterator v605 (http://phages.wustl.edu/starterator/) to refine start coordinates. Putative gene functions were assigned using Phamerator v593 (Cresawn et al., 2011), HHPred (Söding 2005) searches against the the PDB_mmCIF70, Pfam- v.36, NCBI Conserved Domains databases and NCBI Blastp v2.16.0 (Altschul et al. 1990) searches against the Actinobacteriophage and NCBI non-redundant databases. DeepTMHMM v1.0.42 (Hallgren et al. 2022) was used to identify transmembrane domains and tRNAscanSE 2.0 (Lowe & Eddy 1997) to identify tRNAs. All software were used with default settings.

Based on gene content similarity of at least 35% to phages in the Actinobacteriophage database, KNG13 was assigned to actinobacteriophage cluster FE (Pope et al., 2017; Russell & Hatfull, 2016). A total of 26 putative genes were identified, of which 13 could be assigned putative functions. These include several functions related to virion structure and assembly, an endolysin, two proteins with DNA-binding domains, one HNH endonuclease, and a RepA-like replication initiator. Consistent with cluster FE phages, the gene encoding the endolysin is adjacent to two genes that encode transmembrane domains, one of which is assigned as a holin in a subset of phages. The RepA-like replication initiator protein of KNG13 is grouped in a protein family with members only encoded in 4 other FE phages, to date. No lysogeny-related functions could be identified, suggesting KNG13 is unlikely to establish lysogeny.

**Nucleotide sequence accession numbers**

KNG13 is available at GenBank with Accession No. PV876960 and Sequence Read Archive (SRA) No. **SRX31241838** .

## References

[R1] Altschul Stephen F., Gish Warren, Miller Webb, Myers Eugene W., Lipman David J. (1990). Basic local alignment search tool. Journal of Molecular Biology.

[R2] Besemer J., Borodovsky M. (2005). GeneMark: web software for gene finding in prokaryotes, eukaryotes and viruses. Nucleic Acids Research.

[R3] Cresawn Steven G, Bogel Matt, Day Nathan, Jacobs-Sera Deborah, Hendrix Roger W, Hatfull Graham F (2011). Phamerator: a bioinformatic tool for comparative bacteriophage genomics. BMC Bioinformatics.

[R4] Dedrick Rebekah M., Guerrero-Bustamante Carlos A., Garlena Rebecca A., Russell Daniel A., Ford Katrina, Harris Kathryn, Gilmour Kimberly C., Soothill James, Jacobs-Sera Deborah, Schooley Robert T., Hatfull Graham F., Spencer Helen (2019). Engineered bacteriophages for treatment of a patient with a disseminated drug-resistant Mycobacterium abscessus. Nature Medicine.

[R5] Delcher Arthur L., Bratke Kirsten A., Powers Edwin C., Salzberg Steven L. (2007). Identifying bacterial genes and endosymbiont DNA with Glimmer. Bioinformatics.

[R6] Gordon David, Abajian Chris, Green Phil (1998). *Consed:*
A Graphical Tool for Sequence Finishing. Genome Research.

[R7] Hallgren Jeppe, Tsirigos Konstantinos D., Pedersen Mads Damgaard, Almagro Armenteros José Juan, Marcatili Paolo, Nielsen Henrik, Krogh Anders, Winther Ole (2022). DeepTMHMM predicts alpha and beta transmembrane proteins using deep neural networks.

[R8] Hatfull Graham F. (2020). Actinobacteriophages: Genomics, Dynamics, and Applications. Annual Review of Virology.

[R9] Jiang Hongshan, Lei Rong, Ding Shou-Wei, Zhu Shuifang (2014). Skewer: a fast and accurate adapter trimmer for next-generation sequencing paired-end reads. BMC Bioinformatics.

[R10] Lowe Todd M., Eddy Sean R. (1997). tRNAscan-SE: A Program for Improved Detection of Transfer RNA Genes in Genomic Sequence. Nucleic Acids Research.

[R11] Martin Marcel (2011). Cutadapt removes adapter sequences from high-throughput sequencing reads. EMBnet.journal.

[R12] Miller Jason R., Koren Sergey, Sutton Granger (2010). Assembly algorithms for next-generation sequencing data. Genomics.

[R13] Naureen Z, Dautaj A, Anpilogov K, Camilleri G, Dhuli K, Tanzi B, et al., Bertelli M. 2020. Bacteriophages presence in nature and their role in the natural selection of bacterial populations. 10.23750/abm.v91i13-S.10819 PMC8023132 33170167

[R14] Pittsburgh Bacteriophage Institute. 2010. Cluster FE Phages.

[R15] Pope Welkin H., Mavrich Travis N., Garlena Rebecca A., Guerrero-Bustamante Carlos A., Jacobs-Sera Deborah, Montgomery Matthew T., Russell Daniel A., Warner Marcie H., Hatfull Graham F., Science Education Alliance-Phage Hunters Advancing Genomics and Evolutionary Science (SEA-PHAGES) (2017). Bacteriophages of
*Gordonia*
spp. Display a Spectrum of Diversity and Genetic Relationships. mBio.

[R16] Rinehart C, Gaffney B, Wood J, Smith S. 2016. PECAAN, a Phage Evidence Collection And Annotation Network.

[R17] Rogovski Paula, Cadamuro Rafael Dorighello, da Silva Raphael, de Souza Estêvão Brasiliense, Bonatto Charline, Viancelli Aline, Michelon William, Elmahdy Elmahdy M., Treichel Helen, Rodríguez-Lázaro David, Fongaro Gislaine (2021). Uses of Bacteriophages as Bacterial Control Tools and Environmental Safety Indicators. Frontiers in Microbiology.

[R18] Russell Daniel A, Hatfull Graham F (2016). PhagesDB: the actinobacteriophage database. Bioinformatics.

[R19] Science Education Alliance. . Phage Discovery Guide.

[R20] Sharp Richard (2001). Bacteriophages: biology and history. Journal of Chemical Technology & Biotechnology.

[R21] Soding J., Biegert A., Lupas A. N. (2005). The HHpred interactive server for protein homology detection and structure prediction. Nucleic Acids Research.

[R22] Wetzel Katherine S., Aull Haley G., Zack Kira M., Garlena Rebecca A., Hatfull Graham F. (2020). Protein-Mediated and RNA-Based Origins of Replication of Extrachromosomal Mycobacterial Prophages. mBio.

[R23] Wick Ryan R., Judd Louise M., Gorrie Claire L., Holt Kathryn E. (2017). Unicycler: Resolving bacterial genome assemblies from short and long sequencing reads. PLOS Computational Biology.

[R24] Zorawik Michelle, Jacobs-Sera Deborah, Freise Amanda C., Reddi Krisanavane, SEA-PHAGES (2024). Isolation of Bacteriophages on Actinobacteria Hosts. Methods in Molecular Biology.

